# Synthesis and Characterization of Oxidovanadium(IV) Complexes of 2-((E)-(6-Fluorobenzo[d]thiazol-2-ylimino)methyl)-6-methoxyphenol and Their Antimicrobial, Antioxidant, and DNA-Binding Studies

**DOI:** 10.1155/2018/2452869

**Published:** 2018-06-27

**Authors:** K. Savithri, H. D. Revanasiddappa

**Affiliations:** Department of Studies in Chemistry, University of Mysore, Manasagangotri, Mysuru, Karnataka 570 006, India

## Abstract

Two novel oxidovanadium(IV) complexes with a new bidentate (O- and N-) imine-based ligand 2-((E)-(6-fluorobenzo[d]thiazol-2-ylimino)methyl)-6-methoxyphenol (HL) were synthesized under in situ experimental condition where VOSO_4_ acts as a kinetic template in the ratio 2 : 1 (L : M) and mixed ligand complex using 1,10-phenanthroline (phen) in 1 : 1 : 1 (L : M : phen) ratio. The synthesized compounds were structurally characterized by microanalysis, magnetic susceptibility, FTIR, electronic spectra, TG/DTA, ESR, and molar conductance studies. Based on the spectral studies, the complexes have the general composition [VO(L)_2_] (C_**1**_) and [VO(L)phen] (C_**2**_) in a square pyramid geometrical fashion. The synthesized compounds were primarily screened for their *in vitro* growth inhibiting activity against different strains of bacteria, namely, *E. coli*, *B. subtilis*, *S. aureus*, and *P. aeruginosa* by the disc diffusion method. Also, the antifungal activity was determined against *C. albicans* and *A. niger* by the Bateman poisoned technique. The *in vitro* antioxidant activity of all the compounds was determined by DPPH free radical-scavenging assay. Intercalative mode of DNA-binding properties of the oxidovanadium(IV) complexes with calf-thymus DNA (CT-DNA) was investigated using UV, fluorescence spectra, and viscosity measurements.

## 1. Introduction

Vanadium compounds, especially the coordination chemistry of oxidovanadium(IV), have aroused significant interest for several main reasons in recent years especially in the designing of long-acting drugs in metabolism. Research interest in oxidovanadium chemistry derives from its utility in several biological and industrial processes like antimicrobial, spermicidal, antioxidant, antitumor, DNA binding [1], and recently as insulin mimetic [2]. The vital role of vanadium in different chemical and biological systems has motivated the development of vanadium chemistry. The coordination chemistry of nitrogen and oxygen donor ligand is an active area of research. It is evident that the –HC=N linkage in azomethine derivatives is an essential structural requirement for biological activity [3, 4]. The geometry, coordination number, and biological efficacy of oxidovanadium(IV) are highly ligand dependent. It is also known that VO^2+^ is less toxic than the vanadate ion (VO_4_^3−^) and as shown above, with all these characteristics, the complexes exhibit potential therapeutic applications [5].

In addition to that imine bases containing heterocyclic nucleus have potent biological activity.

The *o*-hydroxy imine bases have emerged as an antimicrobial agent of immense interest because of their comprehensive spectrum of *in vitro* and *in vivo* chemotherapeutic action [3, 6]. The metal complexes from bidentate ligand have often been studied recently because of their technical applications in an enhancement of drug action. Transition metal complexes of vanadium with imine bases have been amongst the most widely studied of coordination compounds in the past few years since they are found to be important as analytical and antimicrobial agents. The complexes formed by 3d metals with ligand using both oxygen and nitrogen have been paid close attention in this area.

Antioxidants interfere with the oxidative processes by scavenging free radicals, chelating free catalytic metals, and by acting as electron donors [7]. Interaction study of small molecules with DNA attracts attention for importance in cancer therapy and molecular biology [8]. DNA is involved in many kinds of life processes, and consequently, it is considered to be the prime target of drugs. The transition metal complexes of 1,10-phenanthroline have been widely employed in DNA studies due to their utility in biomedicinal chemistry and in the design of stereospecific DNA-binding drugs [9]. In this perspective, it is essential to know the antioxidant and DNA binding properties of bioactive compounds for their use in the pharmaceutical and therapeutic field.

The current study is structured to provide a synthesis and characterization of a new imine-based ligand (HL) and its oxidovanadium(IV) complexes. The new compounds have been subjected to characterize by different analytical techniques and also exploit some biological applications of oxidovanadium complexes.

## 2. Experimental

### 2.1. Physical Measurements

All the solvents and reagents procured commercially and were used without prior purification. 2-Amino-6-fluorobenzothiazole, 1,10-phenanthroline monohydrate, and *o*-vanillin were purchased from Sigma-Aldrich chemicals, Germany. VOSO_4_·6H_2_O and all other solvents are procured from Merck Specialties Private Limited, Mumbai. The melting point (temperature 0–350°C) of the compounds was determined on an ELICO-3210 apparatus and is uncorrected. FTIR spectra of the compounds were recorded on a PerkinElmer 783 spectrophotometer via attenuated total reflection (FT-ATR) in the range of 4000–400 cm^−1^. Elemental analyses (CHNO) were performed with a model 240 PerkinElmer elemental analyzer. UV-visible spectra of the complexes in the region 200–800 nm were measured using a UV-Vis spectrophotometer (DU 730 “Life Science” M/S Beckman coulter, USA). Molar conductance measurements were performed for the prepared complexes using an Elico CM-180 conductometer with a cell having a cell constant 1. TGA analysis was performed on a Shimadzu AT-50 thermal analyzer from 30 to 900°C with a heating rate at 10°C/min under N_2_ atmosphere. Electron spin resonance (ESR) spectra were recorded using a JEOL JES-TE100 ESR spectrometer in DMSO solution at a liquid-nitrogen temperature on X-band at a frequency of 9.13 GHz under the magnetic field of 300 mT. Ascorbic acid, CT-DNA, and ethidium bromide (EB) were purchased from Sigma Corp., and they were used as received.

### 2.2. Methodology

#### 2.2.1. Synthesis of Imine-Based Ligand (HL)

**Scheme 1:**
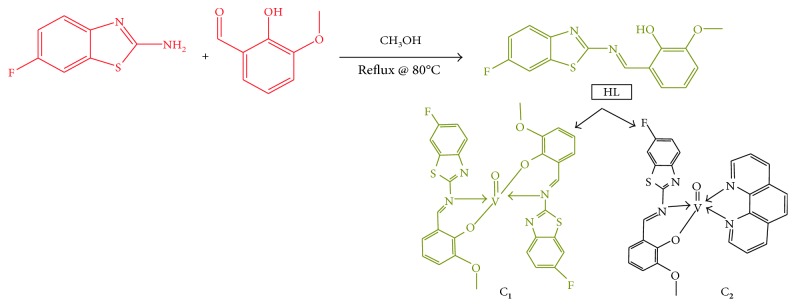
Synthetic route of ligand HL and its complexes.

*(1) Classical Method*. The ligand HL was synthesized by the condensation of 2-amino-6-fluorobenzothiazole with *o*-vanillin in 1 : 1 ratio. To an absolute methanolic solution of 2-amino-6-fluorobenzothiazole (10 mmol, 1.103 g), the methanolic aromatic aldehyde, that is, *o*-vanillin (10 mmol, 1.521 g) was added and the reaction mixture was then stirred under reflux at 80°C for 12 h. The product formed after slow evaporation of the solvent was then isolated by filtration, washed, and recrystallized from methanol. Yield = 78–79%, and synthetic pathway of ligand HL and its complexes is displayed in Scheme 1.

*(2) Microwave Method*. The title compound HL was synthesized by placing an equimolar ratio of the mixture of 2-amino-6-fluorobenzothiazole, and 2-hydroxy-*m*-anisaldehyde was mixed thoroughly in a grinder. The reaction flask was then irradiated in a microwave oven for 7 min using dry methanol (3–4 mL) as a solvent. TLC was used to assess the completion of the reaction progress and to preliminary check on the purity of the product, and the reaction mixture was allowed to attain room temperature (25 ± 2°C). The yellow solid was obtained after recrystallization from methanol, and yield = 87–89%.

*(3) HL: 2-((E)-(6-Fluorobenzo[d]thiazol-2-ylimino)methyl)-6-methoxyphenol*. Mp = 170–172°C; *R*_f_ 0.55, Anal. (%) for C_15_H_11_FN_2_O_2_S: Found (calc.): C 54.80 (54.82), H 2.68 (2.63), N 9.11 (9.13), O 5.21 (5.22); *m*/*z* 302.05 Da; FTIR (υ_max_/cm^−1^); (υOH) 3253, (HC=N) azomethine 1662; ^1^H NMR (295 K/*δ* in ppm/DMSO-*d*_6_ 400 MHz): HC=N 9.776 (1H, s); phenolic OH 11.45 (1H, s); Ar-H 7.0–7.9 (6H, m); ^13^C NMR (295 K/*δ*(ppm)/DMSO-*d*_6_ 400 MHz): 164.176, 123.880, 109.423, 158.848, 109.150, 148.414, 135.710, 161.261, 119.463, 159.63, 115.714, 135.323, 124.494, 129.215.

#### 2.2.2. Synthesis of Complexes

*(1) Preparation of Complex* ***1*** *[C*_***1***_ *]*. The metal salt (VOSO_4_·6H_2_O) (2 mmol) was dissolved in 10 mL of aqueous alcoholic solution, ligand HL (4 mmol) was mixed in 15 mL ethanol in the ratio of 1 : 2, and the reaction mixture was refluxed for 9 h on a water bath. After a specified period of reaction, the solvent was evaporated slowly by allowing standing at room temperature. The resultant yellow color complex was used for further characterization.

*(2) Preparation of Complex* ***2*** *[C*_***2***_ *]*. Complex **2** was prepared by mixing an equimolar ratio of metal salt (VOSO_4_·6H_2_O) in the aqueous medium and an ethanolic solution of ligand HL, and the mixture was stirred for 10 min. 1,10-Phenanthroline monohydrate dissolved in ethanol was added at the same temperature. It was then kept under reflux for 8 h on a water bath. Then, the solvent was evaporated at RT, and the obtained green color complex was used for further analysis.

*(3) C*_***1***_ *: [VO (L*_*1*_)_*2*_*]*_*2*_. Yield = 65%, yellow color, Anal. (%) for C_30_H_20_VF_2_N_4_O_5_S_2_; Found (Calc.) C 53.81 (53.02), H 3.01 (3.12), V 7.61 (7.59), N 8.37 (8.21), O 11.95 (12.05); mp ≥ 300°C. *m*/*z*: 669.02 Da; IR (υ_max_/cm^−1^); (HC=N) azomethine 1640; M-O 542; M-N 463; 362, H_2_O 3387.

*(4) C*_***2***_ *: [VO(L*_*1*_ *)(phen)]*. Yield = 78%, green color, Anal. (%) for C_27_H_18_VFN_4_O_3_S: Found (Calc.) C 59.13 (58.71), H 3.31 (3.14), V 9.29 (9.12), N 10.22 (10.55), O 8.75 (8.99); mp ≥ 300°C. *m*/*z*: 548.461 Da; IR (υ_max_/cm^−1^); (HC=N) azomethine 1633; M-O 576; M-N 468.

### 2.3. Bioassay Studies

#### 2.3.1. Antimicrobial Activity

The biological activities of the compounds have been investigated to display the possibility of their uses in the medicinal field. The *in vitro* antimicrobial screening effects of the synthesized compounds were tested against four bacterial strains, namely, *E. coli*. (MTCC 443), *B. subtilis* (MTCC 441)*, S. aureus* (MTCC 96), and *P. aeruginosa* (MTCC 424) and two fungal species (*A. niger* MTCC 282 and *C. albicans* MTCC 227). The disc diffusion method using the nutrient agar medium for antibacterial activity [10] and Czapek's agar nutrient medium was used for antifungal activity by the Bateman poisoned technique [11]. The bacteria and fungi were subcultured in the sterile autoclaved medium and were incubated up to 24 h for bacteria (37 ± 2°C) and 72 h for fungi at 22 ± 1°C. Standard antibacterial drug chloramphenicol and antifungal drug fluconazole were used for comparison. The stock solution (1 mg/mL) of the test compounds was prepared in DMSO. The antibiotic activity of each compound was evaluated at three different concentrations on dilution of stock solution, namely, 10, 50, and 100 *µ*g/mL. The discs having a diameter of 5 mm were soaked in the test solutions of the above three concentrations and were placed on an appropriate nutrient medium previously seeded with organisms in the Petri dishes and stored in an incubator at the abovementioned period. In order to clarify any effect of DMSO on the biological screening, separate studies were carried out with solutions alone of DMSO and they showed no activity against any microbial strains. The colony diameter of the test organism was measured with the mm scale after a week. The percentage inhibition of the growth of the test organisms was calculated by the Vincent equation:

(1) $$\begin{array}{c} \text{growth inhibition percentage}=\frac{100\left(C_{d}-T_{d}\right)}{C_{d}}, \end{array}$$

where *C*_d_ = colony diameter of the control plate and *T*_d_ = colony diameter of microbial growth on the test plate.

Biocidal activity was assessed by measuring minimum inhibitory concentrations (MICs). The MIC value of each compound was determined at its lowest concentration (highest dilution) required to arrest the growth of microbes by the standard broth dilution assay. Compounds showing promising antibacterial/antifungal activity were selected for minimum inhibitory concentration (MIC) studies.

#### 2.3.2. Time-Dependent Killing Kinetics

Time-dependent killing kinetic analyses of compounds against *E. coli* strain were performed at 0.5×, 1×, and 2× the MIC, as determined previously, and an initial stock culture with a concentration of 1 × 10^6^ CFU per well. After incubation at the time interval of 6, 12, 18, and 24 h at different MICs of the compounds, the absorbance was measured at 625 nm. The culture treated without the compound was kept as the control. The experiment was performed in triplicate. The rate and extent of killing were expressed as mean log_10_·CFU·mL^−1^ against time [12].

#### 2.3.3. Antioxidant Activity

DPPH has an odd electron number with a strong absorption band at 517 nm. When this electron becomes paired off, the absorption decreases stoichiometrically with respect to the number of electrons taken up [7, 13]. Such a change in the absorbance produced in this reaction has been widely applied to test the capacity of various molecules to act as free radical scavengers. Therefore, DPPH is usually used as a substrate to evaluate antioxidant activity of other antioxidants. The present oxidovanadium complexes are in the +4 oxidation state and act as an electron donor compound. It can reduce the DPPH radical to DPPH (*α*,*α*-diphenyl-*β*-picrylhydrazine) compound, and the VO(IV) ion will be oxidized to its +5 state.

The free radical-scavenging activity of the compounds was measured in terms of hydrogen donating or radical-scavenging ability using the stable radical DPPH described by the Blois method [14]. Briefly, stock solutions of the samples (0.001 g/mL) were prepared by dissolving in DMSO. Different concentrations (20–100 *µ*L) of the stock solution were made up to 3 mL of methanol. A solution of DPPH (0.0001 mol) in methanol was prepared, and 1 mL of this solution was added to each of the above test solutions. 0.1 M sodium acetate buffer was added. The mixture was shaken vigorously and incubated for 30 min, and then, the absorbance was measured at 517 nm. All the tests were carried out in triplicate and expressed as the mean ± standard deviation (SD). Ascorbic acid (AA) was used as a standard or positive control, parallel to the test compound and in the absence of the test compound/standard used as the negative control. The capability to scavenge the DPPH radical was calculated using the following equation:

(2) $$\begin{array}{c} I\left(\%\right)=\left(\frac{A_{c}-A_{\text{sample}}}{A_{c}}\right)\times 100, \end{array}$$

where the absorbance of the control reaction mixture excluding the test compounds is termed as *A*_c_ and the absorbance of the test compounds is *A*_sample_.

#### 2.3.4. DNA-Binding Experiments

*(1) Electronic Absorption Spectral Titration*. The stock solution of CT-DNA was prepared in 5 mM Tris-HCl/50 mM NaCl buffer at pH 7, which gave *A*_260_/*A*_280 nm_ of *ca*. 1.8-1.9, indicating that the DNA was sufficiently devoid of protein [15], and the concentration of DNA per nucleotide was determined from its absorption intensity at 260 nm with a known molar absorption coefficient (*ɛ*_260_ = 6600 dm^3^·M^−1^·cm^−1^) [16]. DNA stock solutions were kept stored at 4°C and used within four days. The binding constant (*K*_bin_) values of the complexes to CT-DNA were obtained by monitoring the changes in the absorption of the spectral band intensity with increasing DNA concentration. After equilibrium had been achieved, the spectra were recorded at 200–550 nm against an analogous solution of DNA as a reference. UV spectral data were fitted into the Wolfe–Shimmer equation (3) [17] to obtain the intrinsic binding constant (*K*_bin_)

(3) $$\begin{array}{c} \frac{\left[\text{DNA}\right]}{\varepsilon_{a}-\varepsilon_{b}}=\frac{\left[\text{DNA}\right]}{\varepsilon_{b}-\varepsilon_{f}}+\frac{1}{K_{\text{bin}}\left(\varepsilon_{b}-\varepsilon_{f}\right)}, \end{array}$$

where [DNA] is the concentration of the DNA in base pairs, *ε*_a_ is the apparent extinction coefficient (*A*_obs_/[MC]), *ε*_b_ is the extinction coefficient of the metal complex in the fully bound form, and the extinction coefficient for the free metal complex [MC] is *ε*_f_. The value of intrinsic *K*_bin_ was calculated as the ratio of the slope to intercept. The due correction was made for the absorbance of DNA itself.

*(2) EB Competition Assay*. The competitive binding activity of oxidovanadium complexes with CT-DNA was studied by the fluorescence spectral method using emission intensity of ethidium bromide (EB) with varying concentrations of DNA. EB emits intense fluorescence light in the presence of DNA at *λ*_ex_ 600 nm (546 nm) due to strong stacking intercalation between adjacent DNA base pairs. The fluorescence emission intensity can be quenched when the second molecule (synthesized compounds) was added to the CT-DNA bound EB solution. In the fluorescence quenching study, [DNA]/[EB] = 1.13 is kept constant and varied with the compound concentration. The fluorescence emission spectra of the reaction mixture were recorded between 400 and 850 nm at excitation wavelength 350 nm. The fluorescence quenching efficiency is evaluated according to the following classical Stern–Volmer equation:

(4) $$\begin{array}{c} \frac{I_{o}}{I}=1+k_{\text{sv}}Q, \end{array}$$

where the fluorescence intensities without and with the quencher (complexes) are termed *I*_o_ and *I*, respectively, “*Q*” is the concentration of the quencher, and *K*_sv_ is the linear Stern–Volmer quenching constant. The measured fluorescence intensities were corrected for the dilution and the inner filter effect in the entire DNA-binding experiments.

*(3) Viscosity Measurements*. Viscosity experiments were carried on the Ubbelohde viscometer at a static temperature (26 ± 0.1°C). Compounds were added to the DNA solution (10 *µ*mol·L^−1^) with a micropipette. Flow time was recorded for the increasing concentration of the compounds (0–10 *µ*M). Data were graphically presented as (*η*/*η*_o_)^1/3^ versus the binding ratio of the concentration of compounds to DNA (*r*), where *η* was the viscosity of the DNA in the presence of the compound and *η*_o_ was the viscosity of the DNA alone. The relative viscosity *η* was calculated using the following equation [18]:

(5) $$\begin{array}{c} \eta =\frac{\left(t-t_{o}\right)}{t_{o}}, \end{array}$$

where *t* and *t*_o_ represent the flow time of the buffer solution through the capillary and the observed flow time for the DNA in the presence and absence of the compounds, respectively. The mean of replicate measurements was used to evaluate the viscosity of the samples.

## 3. Results and Discussion

### 3.1. Chemistry

All the synthesized compounds are stable in the atmospheric condition, nonhygroscopic, insoluble in water but slightly soluble in alcohol and readily soluble in DMF and DMSO. Microwave-assisted synthesis of ligand HL was easy, convenient, short reaction period with a good yield (87–89%) and superior to the conventional method (78–79%). Oxidovanadium complexes are highly colored, fine amorphous powder, stable in the solid phase at the ambient temperature in the light. The solutions were stable on keeping for an extended period in the light. The analytical and physical data of the new compounds are summarized in the experimental section. Analytical data show that the complexes have the stoichiometry to be 1 : 2 (metal : ligand) for complex **1**, whereas 1 : 1 : 1 (metal : ligand : phen) for complex **2**. Physical property and analytical data of newly synthesized imine base and its complexes are summarized in the experimental section. The molar conductance values at the 10^−3^·M concentration are too low to account for any dissociation of the complex in DMF and are presented in Table 1. Hence, the synthesized VO(IV) complexes may be regarded as nonelectrolytes. Structural elucidation of the complexes by multispectroscopic techniques reveals that the vanadium center in complex **1** is coordinated to two bidentate imine-based ligands with the two nitrogen and two phenolate oxygen atoms in equatorial positions and oxooxygen in the axial position to complete the square pyramidal N_2_O_3_ coordination sphere. Similarly, N_3_O_2_ coordination sphere can be seen around vanadium in complex **2**.

### 3.1.1. Molar Conductivity Measurements and Magnetic Susceptibility Measurements

The metal complexes were dissolved in DMSO, and the molar conductivities of their solutions at 25 ± 2°C were measured. It is concluded from the results obtained in Table 1, that the complexes have molar conductance value of 10.4–13.9 ohm^−1^·mol^−1^·cm^−1^, indicating that all the complexes are nonelectrolytes. The magnetic susceptibility measurements of the complexes were carried out to find out the effective magnetic moment per each metal in the complexes. The number of unpaired electrons possessed by the metal ion can be determined from the effective magnetic moment of the metal ion. The magnetic susceptibility studies from the literature show that VO(IV) complexes have paramagnetic behavior. Values of magnetic susceptibility (*µ*_eff_) for the VO(IV) complexes are given in Table 1. A mononuclear oxidovanadium(IV) structure is assigned to both complexes **1** and **2**, and the *µ*_eff_ values for these complexes are found to be 1.75 and 1.79 BM, respectively. These values are well suited for complexes of oxidovanadium(IV), *d*^1^ system [19, 20].

### 3.1.2. Electronic Spectra

The electronic absorption spectra of both the complexes were recorded for the freshly prepared solution in DMSO at room temperature over the range 200–800 nm, and the spectral data are listed in Table 1. The electronic spectra of the complexes in which intense absorptions in the region of 328–413 nm are assigned to the spin allowed intraligand (π–π^∗^ and *n*–π^∗^) and ligand-to-metal charge transfer (LMCT) O(π) → V(d) transitions. The weak shoulder appeared around 490 (486–518) and ∼580 nm are due to the d-d transitions ^2^B_2_ (*d*_*xy*_ → *d*_*xz*_, *d*_*yz*_) → ^2^E and ^2^B_2_ (*d*_*xy*_ → *d*_*x*_^2^_−y_^2^) → ^2^B_1_ in vanadium complexes which are consistent with the square pyramidal geometry around the V^4+^ ion [21, 22]. The third absorption is not observed as it might bury beneath the high-intensity charge transfer band. The expected *d*-*d* transition band could not be determined, possibly due to a low concentration of the vanadium(IV) ion in the samples.

### 3.1.3. IR Spectra

The characteristic IR spectral bands of ligand and its complexes are presented in Table 2. The IR spectrum of the ligand shows a strong band at 1037 cm^−1^ region which is assigned to the cyclic–OCH_3_ group of *o*-vanillin. The band for stretching frequency of phenolic OH occurred at 1267 cm^−1^, whereas in IR spectrum of these complexes, this band is shifted to different frequency showing a strong band at around 1258–1271 cm^−1^ region. It indicates that there existed M-O bond formation and it involved in coordination with the metal ion. In the spectrum of the Schiff base, the strong bands at 1666 cm^−1^ region are attributed to -C=N groups in the free ligand. On chelation, due to the possible drift of lone pair electron density towards the metal ion, the azomethine -C=N band is expected to absorb at a lower frequency in the complexes. The band shifted to 1658–1637 cm^−1^ region indicating the coordination of azomethine nitrogen to the metal. Spectra of the complexes also show new peaks at around 413–452 cm^−1^ and 493–552 cm^−1^ region due to the formation of M-O and M-N bands, respectively. In addition to other bands, the oxidovanadium complex shows its characteristic V=O frequency at 980 and 967 cm^−1^ regions for complexes **1** and **2** [23].

### 3.1.4. Thermogravimetric (TG)/Differential Thermogravimetric Analysis (DTA)

**Figure 1:**
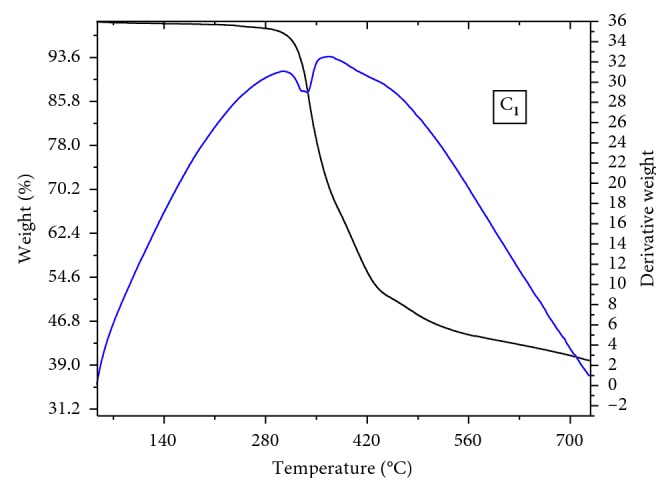
TGA-DTA thermogram of complex **1**.

 of the complexes was carried out under argon flow in the temperature range of 30–900°C. The thermal data of compounds are listed out in Table 3. The thermal stability curve of C_**1**_ (Figure 1) where decomposition starts with loss of two coordinated organic ligands in C_**1**_ in the range of 300–513°C and the associated weight loss found (46.8–97%) is consistent with the calculated values. There is an exothermal peak on the DTA curve in the range of 450–600°C corresponding to the significant weight loss of the ligand moiety of C_**1**_. Similarly, compound C_**2**_ showed two-step of condensation in the thermogram where phenanthroline decomposition occurred at a temperature (105–126°C) lower than that of the ligand (321–394°C). The percentage of the final metal oxide (V_2_O_5_) residue of both the complexes was calculated from the weight of the ash obtained.

### 3.1.5. ESR Spectra

**Figure 2:**
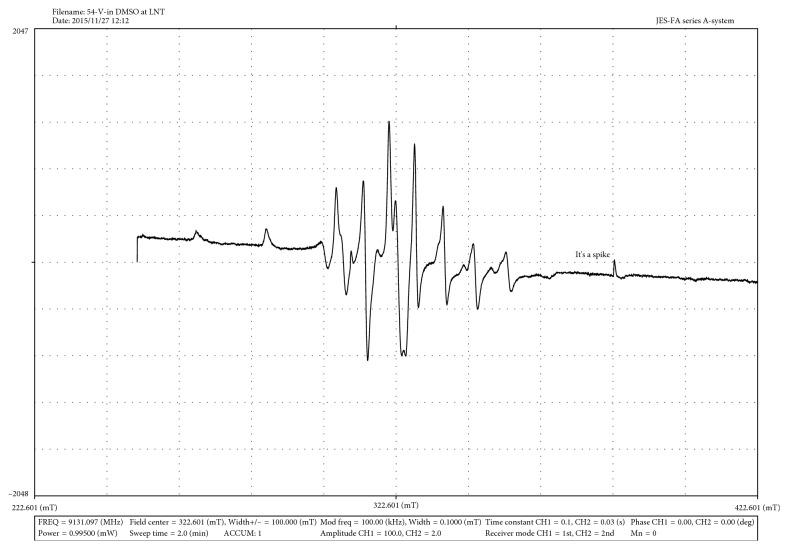
ESR spectrum of C_**2**_ in DMSO at liquid nitrogen temperature.

The complexes C_**1**_ and C_**2**_ were also characterized by ESR spectral measurement recorded at liquid nitrogen temperature in the DMSO solution. The X-band ESR spectra of one representative complex in DMSO at liquid nitrogen temperature are given in Figure 2, whereas each spectrum shows a hyperfine pattern of typical eight equidistant lines originating from the interaction of an unpaired electron with ^51^V, *I* = 7/2 These characteristic spectral features are indicative of monomeric VO(IV) complexes.

The calculated ESR parameters from the spectra of both the complexes are listed in Table 4.

*g*_avg_ corresponds to the isotropic *g*_iso_ value was calculated to be 1.952 and 1.958 for C_**1**_ and C_**2**_, respectively. The parameters showing the orders of *g*_||_ < *g*_⊥_ and *A*_||_ >> *A*_⊥_ indicate that an unpaired electron is present in the (*d*_*xy*_)^1^ orbital with a square pyramidal geometry around the oxidovanadium(IV) complexes in C_4v_ symmetric fashion [5, 24]. Within the limits of errors, the experimental *A*_||_ agrees with the calculated one, suggesting that the solution structure at low temperature does not significantly deviate from the structure in the solid state.

The preceding observations further support the composition of the complexes, which is compatible with the results, obtained by each other methods.

### 3.2. Biology

#### 3.2.1. Antimicrobial Activity

The antimicrobial activities of the HL and its complexes have been investigated to show their impressive level of antimicrobial activity of MIC values against newly emerging highly drug-resistant pathogens. The results of *in vitro* study of the microbicidal activity of the afresh synthesized complexes against four bacterial strains (Gram negative: *E. coli* and *B. subtilis* and Gram positive: *S. aureus* and *P. aeruginosa*) and two fungal strains (*A. niger* and *C. albicans*) are reported in Table 5.

The free ligand HL and its VO(IV) complexes show varying degree of inhibition effects toward all the organisms, bacteria, and fungi. It is evident that the metal coordination tends to make the ligands strong bacteriostatic and fungistatic agents and efficiently inhibits both bacterial cell and mycelial growth of drug-resistant microbes more than the parent ligand. The oxidovanadium(IV) complex C_**2**_ of mixed ligands showed good antifungal activity rather than antibacterial than the free ligand and is almost nearer to the standard drugs used.

The minimum inhibitory concentration (MIC) of compounds, which showed significant activity against bacterial and fungal species, was also determined (Table 6). The MIC of these compounds varies from 10 to 100 mg/mL. The results indicated that these compounds were the most active in inhibiting the growth of the tested species at 10 mg/mL concentration.

These results show the structure activity relationship between the organism and the substituted saline moiety. The inhibition values indicate that most complexes have higher percentage towards Gram-negative bacteria compared to Gram positive is due to the metal ions shared with the donor atoms (N and O) of the ligand and the π-electron delocalization over the chelate ring increases electron density over C=N nitrogen leading to strong interactions with cell constituents. Such increased activity of the metal chelates can be explained on the basis of the oxidation state of the metal ion and chelation theory (Tweedy's and Overtone's concept) [20, 25].

Mechanism of action seems that the structural components possessing additional azomethine linkage with heteroatoms inhibit enzyme activity due to their deactivation by metal coordination. This permits their efficient membrane permeabilizing ability through the lipoid layer of organisms and destroys their activity [26]. The enhanced antimicrobial potency of the complexes may also be explained by their solubility, conductivity, fitness of the particles, cell permeability, size of the metal ion, and bond length between metal and ligand.

Membrane-targeting antibiotics will exhibit charge-based selectivity toward the bacterial membrane. In general, Gram-positive bacteria have a simpler cell wall than Gram-negative bacteria, and the primary constituent is peptidoglycan, a polysaccharide. In contrast, Gram-negative bacteria have an additional outer bilayer membrane composed of lipopolysaccharides and phospholipids [27, 28].

**Figure 3:**
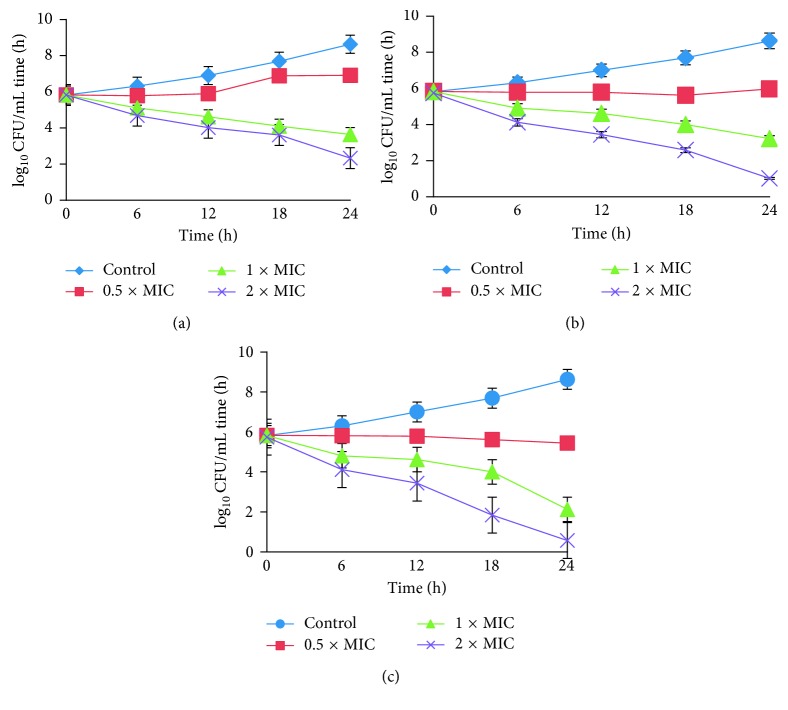
Time-course kinetics of the bactericidal activity of (a) HL, (b) C_**1**_, and (c) C_**2**_ on *E. coli* after exposure to 0.5×, 1×, and 2× the MIC and the control group treated with normal saline.

*(1) Time-Dependent Kinetics*. Killing kinetics exhibited marked effect made by complexes than free ligand on bacterial strain chosen. The control showed no noticeable bactericidal activity throughout 24 h incubation for all the compounds performed. The ligand HL (Figure 3(a)) at 0.5× MIC showed no significant effect on *E. coli* up to 18 h, and after that, it showed a slow decline up to 24 h. At 1× and 2× the MICs, the rate of killing increased with increasing incubation time and reached ∼1 × 10^3^ and ∼1 × 10^2^ CFU·mL^−1^, respectively. Time-kill curve for complex C_**2**_ is displayed in Figures 3(b) and 3(c) against *E. coli*. When a mild activity was observed at 0.5×, the rate of killing was gradually increased with time and complete killing occurred at 2× the MIC. Complexes C_**1**_ and C_**2**_ showed a more or less similar kind of killing kinetics which showed modest activity at 1× and 2× the MIC.

#### 3.2.2. Antioxidant Activity

**Figure 4:**
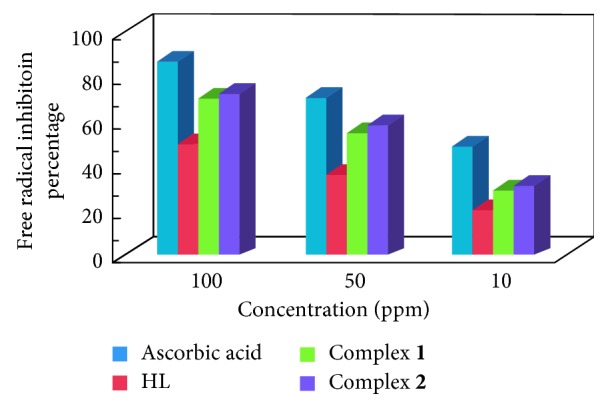
Antioxidant activity of the imine-based ligand (HL) and its metal complexes.

Oxidovanadium(IV) complexes exhibit good DNA-binding affinity, and it is considered worthwhile to study the antioxidant activity of these compounds. Free radicals play a vital role in the pathogenesis of various human diseases and aging. In food products, free radicals also cause damage, resulting in diminishing taste and shelf life. Antioxidants are therefore protecting against free radicals and save health. Mostly, strong antioxidant activity is shown by polyphenols. Our study aimed at exploring the most potent antioxidant and examining the factors that give a picture and establish the antioxidant activity with frequent comparison to different concentrations of HL and its VO(IV) complexes (Figure 4). The results revealed that ligand showed low-scavenging potential (∼20.32–49.78%) in all the screened concentrations; however, upon complexation with oxidovanadium ions, antioxidant activity has been enhanced significantly (∼70–73%) through oxidation of V^(IV)^O^2+^ to V^(V)^O^+^ using water as a medium in the reaction mixture since assay was run in the methanolic system [29]. It is believed that the large conjugated system and chelation of the organic molecules with the metal ions by nitrogen and phenolic hydroxyl group benefits the trapping of free radicals. It is further supported by the observed discoloration from purple DPPH radical solution to yellow solution showing scavenging of the DPPH radicals by hydrogen donation.

#### 3.2.3. DNA Binding Study

*(1) Electronic Absorption Spectral Features of CT-DNA Binding*. Electronic absorption spectroscopy is one of the most useful techniques in DNA-binding studies since the observed changes in the spectra may indicate the mode of interaction.

**Figure 5:**
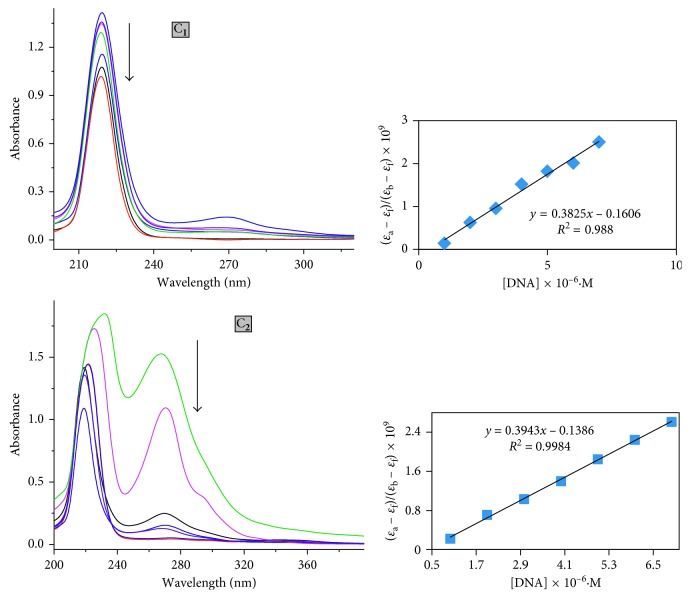
The electronic absorption spectra of complex C_**1**_ and C_**2**_ (20 *µ*M) in the absence and presence of DNA in Tris/NaCl buffer (pH = 7.2). The arrows show the absorbance changes upon increasing concentration of DNA (0–25 *μ*M) at RT. The plot shows [DNA]/(*ɛ*_a_ − *ɛ*_f_) × 10^−5^ versus [DNA].

The UV–Vis titration of both complexes in the absence and in the presence of CT-DNA has been studied in Tris-buffer medium. The absorption spectra of complexes (at a constant concentration of complexes and a wavelength range of 800–200 nm) are given in Figure 5. An increase in the concentration of CT-DNA causes hypochromism for the high-energy absorption intensity band of complex **1** at 218 nm, and on the contrary, the low energy band at 269 nm shows the same with DNA addition. A similar pattern has been observed for the complex **2** at ∼218–231 nm (218, 221, 224, and 231 nm), which exhibits hypochromism and a significant bathochromism of about 12 nm. These spectral characteristics are typical complexes to have higher binding propensity with DNA most likely through a stacking mode of interaction between the planar aromatic chromophore and the DNA base pairs. To further elucidate the binding strength of the complexes, intrinsic binding constants *K*_bin_ were calculated by monitoring the changes of absorbance in the MLCT bands with increasing amounts of CT-DNA. The binding constant (*K*_bin_) values of complexes are quantified from (1), and the values found to be 5.95 × 10^5^, 9.86 × 10^6^ and 5.61 × 10^3^ for complexes C_**1**_, C_**2**_, and HL, respectively. The obtained *K*_bin_ values of the complexes are almost parallel to those reported for the typical classical intercalator ethidium bromide (*K*_bin_, 10^6^–10^7^·M^−1^) and suggests that the binding mechanism of oxidovanadium complexes with DNA is going through the intercalative mode, while the intercalative ability of nonplanar ligand to DNA appears weak and suggests that ligand is involved in DNA groove binding. The relative *K*_bin_ value and pronounced hyperchromism of complexes C_**2**_ in comparison to C_**1**_ with respect to CT-DNA were owing to the planar and larger surface area of the 1,10-phenanthroline ring appended deeply in the complex cause enlargement of size of the DNA.

It also suggested that the DNA-binding affinities of these oxidovanadium complexes are associated with the electronic effects of the substituents introduced on the benzene ring of the ligands. The imine-based ligand would encourage partial intercalation of compounds in the minor groove of DNA helices by engaging in hydrogen-bonding interactions between coordinated –NH– and –OH bands with the functional groups positioned on the edge of DNA bases [30].

*(2) Fluorescence Emission Titrations for DNA Interaction*. Fluorescence quenching tendency is further examined to ascertain the relative binding mode of oxidovanadium complexes with CT-DNA due to high sensitivity and selectivity of this method [31]. The oxidovanadium complexes either in aqueous solution or the presence of CT-DNA do not exhibit any fluorescence emission. So, the interaction of the complexes to DNA has been studied by the competitive ethidium bromide (EB) binding assay.

**Figure 6:**
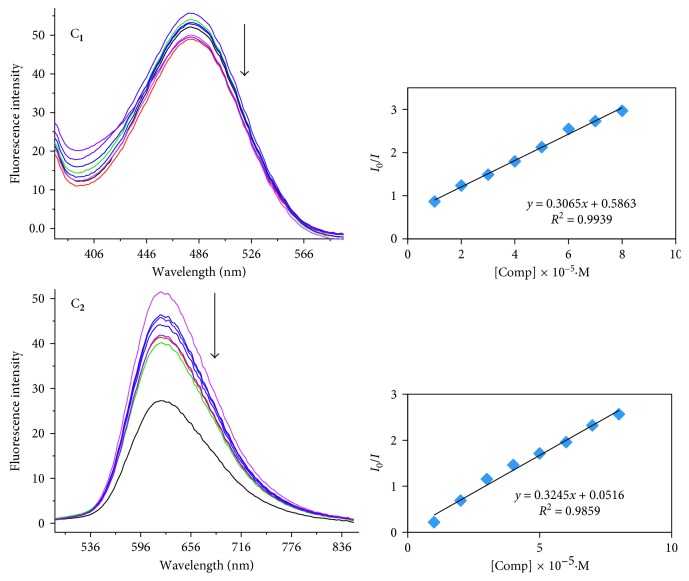
Fluorescence quenching curves of EB-DNA in the presence of complex (C_**1**_ and C_**2**_) at the concentration 0–35 *μ*M at the interval of 5 in Tris-HCl/NaCl buffer (pH 7.2). The arrow indicates the effect of increasing the complex concentration on the fluorescence emission of ethidium bromide bound CT-DNA. The plot shows the linear fit of *F*_o_/*F* versus [complex].

Successive addition of the DNA solution into a constant concentration of complexes led to changes in the fluorescence intensity of complexes. Quenching of fluorescence intensity of compounds was observed and is displayed in Figure 6. The fluorescence spectra of complex **1** showed the emission band at *λ*_em_ 486 nm, and in complex **2**, the emission band was observed at 656 nm when excited at wavelength *λ*_ex_ 350 nm and 620 nm, respectively. The intense fluorescence of the DNA-EB complex system can be quenched by the addition of a third molecule which can bind DNA by the intercalative mode by displacing EB. This significant decrease in fluorescence intensity affords strong support in favor of an intercalative binding pattern of the complex into the DNA double helix.

Furthermore, the fluorescence quenching study in the presence of the complexes was analyzed by the Stern–Volmer quenching constant (*K*_sv_). The obtained SV plots are linear (Figure 6), and the values are found to be 1.09 (±0.02) × 10^4^·M^−1^ for complex **1** and 3.25 (±0.01) × 10^4^·M^−1^ at 37°C. Hence, the fluorescence quenching has static mechanism as the fluorophore, and the quencher collides together in the ground state. The values of *K*_q_ obtained for DNA was about 10^12^·M^−1^·S^−1^. This again proves that the fluorescence quenching of the biomolecule occurs by the static mode since *k*_q_ values are higher than limiting diffusion constant of the diffusional quenching for biopolymers (2∗10^11^·M^−1^·S^−1^).

**Figure 7:**
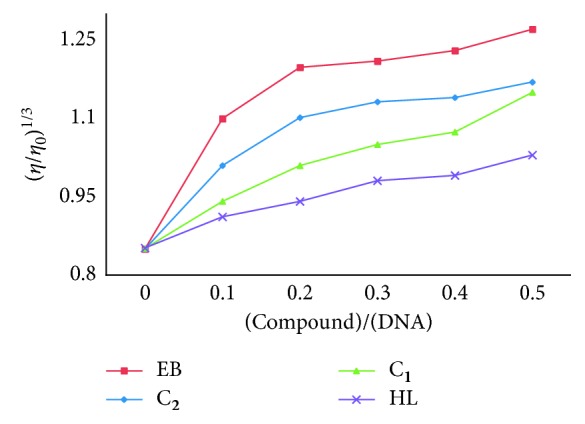
Effect on relative viscosity (±0.1) of CT-DNA under the influence of increasing amount of compounds at 26 ± 0.1°C in 5 mM Tris-HCl buffer (pH 7.2).

*(3) Viscosity Measurements*. The most critical, least ambiguous, and effective means to analyze the binding mode of compounds to DNA is the viscometry technique in solution can be carried out in the absence of X-ray structural data. A classical intercalation probe (EB) between the two strands of DNA results in significant increase in viscosity of the DNA solution due to an increase in separation of base pairs at the intercalation sites and hence, an increase in overall DNA length. In contrast, partial, nonclassical intercalation of ligand could crook the DNA helix, resulting in shortening of DNA length and, concomitantly, reducing its viscosity [32]. The thickness of CT-DNA increases with increase in the ratio of complexes to CT-DNA is associated with the specific intercalation binding mode [28]. Also, the effects of relative viscosity of CT-DNA under the influence of increasing amounts of the complexes at 26 ± 0.1°C (Figure 7) resemble the binding mode of ethidium bromide to DNA. It can be deduced that the obtained results were parallel to the above UV-spectroscopic data such as hypochromism and red-shift of complexes in the presence of DNA.

## 4. Conclusion

In the present investigation, the synthesis, spectral characterization, *in vitro* biopotency studies of VO(IV) complexes of imine-based ligand (HL) were carried out. Elemental analysis confirms the stoichiometry in complexes. Physical properties like molar conductivity, IR, magnetic susceptibility, and electronic spectral properties gave satisfactory analytical data coupled with ESR, and TGA studies suggested the nature of coordination for both the oxidovanadium(IV) complexes to be square pyramidal geometry with point group C_4V_. The synthesized compounds had a broad spectrum of antimicrobial activity against a panel of highly resistant G −ve, G +ve, and fungal pathogens which showed that the complexes have better antibiotic action than its parent ligand and standard drugs used. The antioxidant activity of the complexes found to be as a good scavenger of DPPH radical comparing to free ligand. Spectral titration assessed that the metal complexes strongly bind with DNA relative to the ligand, and the results are corroborated with each other techniques.

## Figures and Tables

**Table 1:** Electronic spectra and magnetic moments of the metal complexes.

Compounds	*µ*_eff_ (BM)	*Λ* (Ohm^−1^·cm^2^·mol^−1^)	Electronic spectral bands (nm)	
			MLCT	*d*-*d* transition
C_**1**_	1.75	13.9	425	555
C_**2**_	1.79	10.4	435	565

**Table 2:** Important infrared frequencies (cm^−1^) of HL and its complexes.

Complexes	ν (C=N)	ν (pH-OH)	ν (OCH_3_)	ν (V=O)	ν (M-N)	ν (M-O)
HL	1666	1267	1019	—	—	—
Complex **1**	1658	1271	1083	980	544	448
Complex **2**	1653	—	1025	967	507	452

**Table 3:** TGA analysis of synthesized compounds.

Serial number	Compounds	Stages	Temperature range (TG) (°C)	Probable composition of expelled group/s	Weight loss found (%)		No. of molecules	% residue	Nature
					Cal.	Expt.			
1	C_**1**_	I	302–513	HL	91.2	93.3	2	8.7	V_2_O_5_

2	C_**2**_	I	105–126	1,10-phen	24.4	23.6	1	12.6	V_2_O_5_
		II	321–394	HL	47.0	46.2	1		

**Table 4:** ESR spectral data of oxidovanadium(IV) complexes in DMSO at liquid nitrogen temperature.

Complexes	*g*_||_	*g*_⊥_	*g*_avg_	*A*_||_ (×10^4^·cm^−1^)	*A*_⊥_ (×10^4^·cm^−1^)
C_**1**_	1.9302	1.9736	1.952	158.8	53
C_**2**_	1.9310	1.9864	1.958	154.6	54

**Table 5:** Antimicrobial results of HL and its metal complexes.

Serial number	Compounds (30 *µ*g/disc)	% growth inhibition in *µ*g/mL concentration																	
		Gram-negative bacteria						Gram-positive bacteria						Fungi					
		*E. coli*			*P. aeruginosa*			*B. subtilis*			*S. aureus*			*C. albicans*			*A. niger*		
		100	50	10	100	50	10	100	50	10	100	50	10	100	50	10	100	50	10
1	HL	16.3	14.8	9.3	48.6	37.1	28.2	47.3	35.3	27.6	23.6	16.7	10.5	40.2	36.6	34.3	41.3	35.5	30.2
2	C_1_	67.2	52.2	42.1	66.7	51.8	38.2	68.6	54.6	42.2	67.4	51.1	46.3	79.3	57.5	36.5	62.1	54.3	39.4
3	C_**2**_	73.8	46.3	36.9	61.3	45.2	32.5	77.5	63.2	36.4	73.7	43.7	32.6	70.2	53.6	41.1	87.6	42.5	41.8
4	Ciprofloxacin	96.4	88.2	69.6	85.8	72.9	50.8	92.5	74.6	66.5	91.6	79.5	61.2		—			—	
5	Fluconazole	—			—			—			—			98.6	81.6	72.3	88.2	80.3	66.3
6	Negative control (DMSO)	100			100			100			100			100			100		

Each value was replicated thrice.

**Table 6:** Minimum inhibitory concentration (mg/mL) of compounds against bacteria and fungi.

Serial number	Compounds	*E. coli*	*P. aeruginosa*	*B. subtilis*	*S. aureus*	*C. albicans*	*A. niger*
1	HL	15	25	30	15	30	22
2	C_**1**_	20	15	10	10	20	30
3	C_**2**_	10	20	15	20	10	10

## Data Availability

The data used to support the findings of this study are available from the corresponding author upon request.
